# Expression and Purification of Recombinant Mycobacterium Tuberculosis (TB) Antigens, ESAT-6, CFP-10 and ESAT- 6/CFP-10 and Their Diagnosis Potential for Detection of TB Patients

**Published:** 2011-08-01

**Authors:** M Hemmati, A Seghatoleslam, M Rasti, S Ebadat, N Mosavari, M Habibagahi, M Taheri, A R Sardarian, Z Mostafavi-Pour

**Affiliations:** 1 Department of Biochemistry, Recombinant Protein Laboratory, Medical School, Shiraz University of Medical Sciences, Shiraz, Iran; 2 Department of PPD Tuberculin, Razi Vaccine and Serum Research Institute, Tehran, Iran; 3 Department of Immunology, Medical School, Shiraz University of Medical Sciences, Shiraz, Iran; 4 Department of Paramedical Sciences, Shiraz University of Medical Sciences, Shiraz, Iran; 5 Dental School, Shiraz University of Medical Sciences, Shiraz, Iran; 6 Department of Biomedical Sciences, Shiraz University of Medical Sciences, Shiraz, Iran

**Keywords:** Mycobacterium tuberculosis, ELISPOT, CFP-10, ESAT-6, Tuberculosis, Diagnosis

## Abstract

**Background:**

One of the most widely used methods to detect tuberculosis (TB) infection is the tuberculin skin test (TST). The completion of Mycobacterium tuberculosis (M. tuberculosis) genome sequence has led to identification of several antigens that can be utilized for accurate diagnosis and control of TB. The aim of this study was to purify the recombinant M. tuberculosis antigens for the evaluation of their potential in TB diagnosis.

**Methods:**

The recombinant secretory antigens, ESAT-6, CFP-10 and ESAT-6/CFP-10 were produced by PCR and cloning methods. To investigate antigen specific responses of these recombinant antigens in detection of TB, ex vivo enzyme linked immunospot (ELISPOT) test in 30 clinically diagnosed TB patients was evaluated.

**Results:**

The selected M. tuberculosis antigens were cloned, expressed and purified in Escherichia coli (BL21). ELISPOT assay for detection of TB showed the sensitivity of 93, 90 and 100% for recombinant ESAT-6, CFP-10 and ESAT-6/CFP-10 proteins respectively, which is significantly higher than conventional TST.

**Conclusion:**

The recombinant antigens of ESAT-6, CFP-10 and ESAT-6/CFP-10 can be used as an accurate means of detecting TB in Iran.

## Introduction

Despite more than a century of research on detection and treatment of Tuberculosis (TB) infection throughout the world, the disease remains a major cause of morbidity and mortality. TB accounts for approximately three million deaths annually with nine million new cases of the disease diagnosed each year.[1][2] In 2008, the World Health Organization (WHO) estimated that yearly about 1.7 million people die because of TB.[3] The incidence of all forms of TB in Eastern Mediterranean sub region countries such as Iran is 104 per 100000 and the incidence of cases which were smear positive is 24 per 100000 populations. [4] In Iran, most of the new TB cases occur among immigrants especially in Afghan immigrants.

The major cause of TB is Mycobacterium tuberculosis (M. tuberculosis) which is an intracellular pathogen. Alveolar macrophages are the main target of invasion by M. tuberculosis bacilli that have the potential to survive and grow in this cells.[5] Fast and accurate diagnosis of TB is an important element in controlling the disease. During the recent years, there has been emphasis on the development of new and rapid tests for the diagnosis of TB such as detection of M. tuberculosis components in clinical specimen using cell-mediated immune (CMI) responses.[6]

Tuberculin skin test (TST) with purified protein derivative (PPD) as one of the CMI-based tests in detection of TB infection has low specificity.[7] Therefore, a new diagnostic test based on specific antigens would be of great value in screening and detection of TB. The availability of the completely sequenced genome of M. tuberculosis has provided tools for the identification of bacterial antigens that are useful in the development of new reagents to diagnose and control TB.[7]

Genome of M. tuberculosis consists of 16 regions of differences (RD). Among these regions, the RD1 locus which plays a key role in the virulence of M. tuberculosis, is present in clinically pathogenic strains of M. tuberculosis and M. bovis, but is deleted in M. bovis BCG (bacillus Calmette-Guerin) vaccine strains.4 This region contains nine protein coding genes (RV3871-3879c), which probably encodes protective and/or virulent antigens.[7][8]

Among the major antigens of RD1 locus, early secretory antigenic target-6 (ESAT-6) and culture filtrate protein-10 (CFP-10) are encoded by the genes esxA and esxB respectively and form a 1:1 heterodimeric complex in vitro. ESAT-6 is the bestcharacterized protein within the RD1 region. It has been recognized as an important stimulator of T-cells both in vitro and in vivo.[9] Also, it has been proposed as a tool for diagnosis of M. tuberculosis infection and frequently used in enzyme-linked immunospot assay (ELISPOT).[10][11] This assay is a specific method for identifying M. tuberculosis infection from the response to BCG vaccination. It has based on production of interferon gamma (IFN-γ by activated T cells after exposure to M. tuberculosis antigens.

The CMI-based tests such as ELISPOT permit early detection of TB infection in a setting of low endemicity and provide an inexpensive tool for diagnosis of TB in developing countries. Given the significance of ESAT-6 and CFP-10 secretion in mycobacterial virulence and the importance of these antigens as diagnostic tools for the detection of TB, we expressed and purified these two antigens as well as their fused form. In order to evaluate their validity for detection of TB patients, commercial ESAT-6 was used and its diagnostic potential compared with the produced recombinant proteins.

## Materials and Methods

The study population (n=60) was divided into two groups: The first group (n=30) comprised of patients who were diagnosed with pulmonary TB (as confirmed by examination of sputum for acid-fast bacilli and/or culturing), with no history of treatment for the disease. Twenty percent of these patients were Afghan immigrants and PPD skin test for all TB patients was performed. Twenty five percent of the patients had negative PPD skin test. All blood samples were obtained from the newly diagnosed sputum smear positive patients reported to the Masih Daneshvari Hospital in Tehran before administration of chemotherapy. The healthy BCG-vaccinated individuals (n=30) were group number 2. Healthy donors were individuals without prolonged direct contact with TB patients, with no vaccination during 10 years ago or any other clinical TB symptom. HIV positive individuals were excluded from the study. All the patients and normal subjects gave their written informed consent prior to participation in the study.

Mouse monoclonal ESAT-6 antibody and Glutathione S Transferase (GST) antibody were obtained from Santa Cruz Biotechnology (Santa Cruz, CA) and mouse monoclonal CFP-10 antibody was purchased from Abcam; Glutathione agarose was purchased from Sigma and isopropyl-1-thio-β-D-galactopyranoside (IPTG) was obtained from Fermentas (USA). Commercial ESAT-6 was obtained from Staten Serum Institute (Copenhagen, Denmark).

Purified DNA from different strains of Mycobacteria including Mycobacterium tuberculosis C (M. tuberculosis C), Mycobacterium tuberculosis DT, Mycobacterium tuberculosis PN, Mycobacterium bovis AN5 and Mycobacterium IR 150 were provided from Razi Serum and Vaccine Research Institute (Tehran, Iran). Using bioinformatic analysis, we compared the genome of these strains with M. tuberculosis H37RV. Native M. tuberculosis C strain, consequently was selected for our analysis due to its genomic resemblance to H37RV. Standard procedures for PCR, transformation, cloning and analysis of DNA were used.[5][6][7][8][9][10][11][12] All constructs made by PCR were sequenced to verify their integrity. Esat-6, cfp-10 and esat-6/cfp-10 fusion genes were amplified from genomic DNA of M. tuberculosis C using the forward and reverse primers shown in Table 1. The PCR products were cloned into pGEX4T1 containing N-terminal GST tag. Recombinant plasmids were transformed to E. coli (DH5α). Positive clones were evaluated to find the correct insertion.

Expression and purification of the recombinant proteins were performed by standard protocols.[5][7] E. coli BL21 (DE3) transformed by pGEX4T1 (+) was grown in LB medium containing 100 μg/ml ampicillin. Expression was induced at mid-log phase by adding IPTG. Cell pellets were lyzed in lysis buffer comprises of 1% of Triton X-100, 1 mM EDTA (pH=8.0), aprotinin, leupeptin and pepstain A (1 μg/ml of each) in PBS. The cell lysates were sonicated, and the suspension was centrifuged.[5] The mixture was allowed to bind to glutathione agarose beads. The lysate-bead mixture was incubated with reduced glutathione reagent to elute GSTtagged proteins. After dialysis, purity of proteins was analyzed by SDS-PAGE and western blotting. For producing pure recombinant proteins without GST-tag, thrombin cleavage of the proteins was performed.

The SDS-PAGE of recombinant protein products was performed in the presence of 0.1% SDS in the gel. Protein samples (with and without GST) were prepared by mixing with reducing sample buffers13 and boiling in a sand bath for 5 minutes. Gel was run at a constant voltage of 200 V. Recombinant proteins transferred to nitrocellulose membranes (Millipore, Biomanufacturing and Life Science Research) using mini Trans-blot Cell (Bio-Rad). The blots were incubated with an appropriate dilutions of primary anti-ESAT-6, anti-CFP-10 and anti-GST antibodies and then were incubated with horseradish peroxidase-conjugated secondary antibody solution. Finally blots were processed using Enhanced chemiluminescence (ECL).[5]

The Far-UV CD spectra were used to determine the secondary structure of the recombinant proteins in an Avir circular dichroism spectropolarimeter model 215. Protein samples were dissolved in buffer 50 mM Tris-HCl pH 8.0, 180 mM NaCl, 1 mM Dithiothreitol (DTT) and 10% v/v glycerol. CD Spectra of the proteins were recorded from 190 to 260 nm, each spectrum representing an average of 3 accumulations.

Interferon gamma ELISPOT assay for detection and enumeration of IFN-γ producing T-cells against mycobacterial antigens was performed by using a commercially available anti IFN-γ antibody pair and black spot developing reagents from U-Cytech (U-CyTech, Netherland). [11][12] Briefly, peripheral blood mononuclear cells (PBMCs) (2×10(5) cells/well) were added to duplicate wells pre-coated with anti- IFN-γ antibody. Following the overnight stimulation with ESAT-6, CFP-10, ESAT- 6/CFP-10, commercial ESAT-6 and PPD, plain medium (as negative control), and phytohemagglutinin (PHA, as positive control), the wells were washed, and the presence of IFN-γ was detected by biotinylated anti-IFN-γ and φ-labeled goat anti-biotin antibody. Afterward addition of activators I and II precipitated silver on φ and revealed the spots of IFN-γ secreting cells. The spots were enumerated with a stereomicroscope and photographed digitally. The number of spots in the back ground control wells was subtracted from the number in the test wells and a response was considered positive if the number of spots per test well was greater than the cutoff point for each antigen and at least twice the value found in the background control wells. The means of the duplicates were used and all results were expressed as number of IFN-γ spot-forming cells (SFC) per million PBMC for comparison.[14][15]

The statistical significance of differences between experimental and control groups were determined by Mann-Whitney U test (SPSS software, version 16.0, Chicago, IL, USA). Data were considered statistically significant at p<0.05. For evaluation of antigen responses, cutoff values were calculated for each antigen as the means of spot forming cells + two standard deviation (SD) obtained with the PBMCs from 30 healthy BCG-vaccinated donors.

**Table 1:** Primers and vector used for cloning of esat-6, cfp-10 and esat-6/cfp-10^a^

**Antigen**	**Primer sequence ( 5' to 3' )**		**Forward (F) or Reverse (R)**	**Cloned between**	**Vector**
ESAT-6	TAA**GGATCC**ATGACAGAGCAGGAGTG		F	BamHI and ECORI	pGEX4T1
	GC**GAATTC**TGCGAACATCCCAGTG		R		
CFP-10	TAA**GGATCC**ATGGCAGAGATGAAGAAC		F	BamHI and ECORI	pGEX4T1
	GC**GAATTC**GAAGCCCATTTGCGAGGA		R		
	TAA**GGATCC**ATGACAGAGCAGGAGTG		F	BamHI	pGEX4T1
ESAT-6 /CFP-10	GGAACCTGGAGA TGCGAACATCCCAGTG		R		
	TCTCCAGGTTCC ATGGCAGAGATGAAGAAC		F		
	AAG**GAATTC**GAAGCCCATTTGCGAGGA		R	ECORI	pGEX4T1
	**GGATCC** : BamHI site, **GAATTC** :ECORI site				
	GGAACCTGGAGA: Linker TCTCCAGGTTCC: linker				

^a^ Restriction sites are underlined.

## Results

In this study, we amplified esat-6, cfp-10 and esat- 6/cfp-10 fusion genes from native M. tuberculosis C genomic DNA by PCR. All PCR products, 288 bp for esat-6, 303 bp for cfp-10 and 591 bp for esat6/cfp10 were cloned into pGEX4T1 expression vector. DNA sequencing of the genes showed that all cloned genes were identical to genes reported on gene bank (esat-6, Accession Nos.RV3875; cfp-10, Accession Nos. Rv3874). Esat-6 and cfp-10 genes were completely inserted in the fusion gene.

**Fig. 1:**
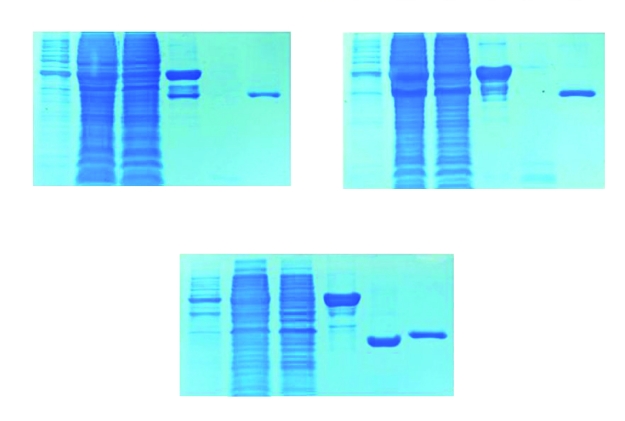
Expression and purification of ESAT-6, CFP-10 and ESAT-6/CFP-10 proteins. Samples (Lane 1-6) were resolved in a 16% (w/v) polyacrylamide gel, under reducing conditions. 1) Un-induced cell; 2) induced cell; 3) glutathione-agarose flow through (unbound); 4) washed glutathione agarose showing bound protein; 5) thrombin cleaved supernatant; 6) washed glutathione agarose after thrombin cleavage. Molecular weight standards are shown to the left of lane 1.

**Fig. 2:**
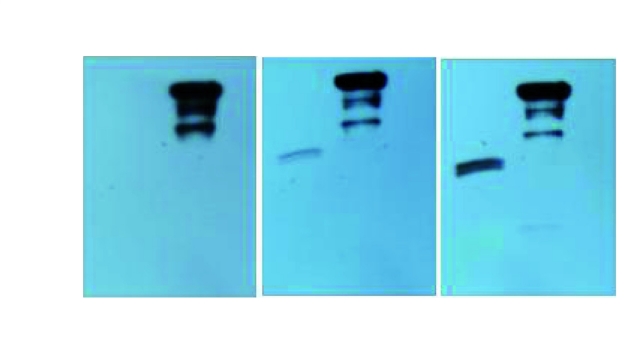
Western blots of ESAT-6/CFP-10 fusion protein probed with mouse anti-GST (A), mouse anti-ESAT-6 (B), and mouse anti-CFP-10 (C) monoclonal antibodies. Lanes1and 2 represent ESAT-6/CFP-10 protein with and without thrombin cleavage.

The recombinant proteins were all purified from cell-free supernatants by affinity chromatography on GST-Agarose. The expressed products were subjected to SDS-PAGE using mini format vertical electrophoresis (BIO-RAD, USA). Gels were run in tricinetris running buffer. Expression and purification results for ESAT-6, CFP-10 and ESAT6/CFP10 proteins were shown in Figure 1. The theoretical molecular weight of pure ESAT-6, CFP-10, and ESAT- 6/CFP-10 is about 10, 13 and 25 KDa, respectively in E. coli expression system. The resolved proteins were transferred to nitrocellulose membrane to identify expression, by western immunoblotting using mini trans–blot cell (Bio-Rad, USA). ESAT-6/CFP-10 fusion protein could be recognized by mouse anti GST, anti ESAT-6 and anti-CFP-10 monoclonal antibodies. Western blot results were shown in Figure 2.

**Fig. 3:**
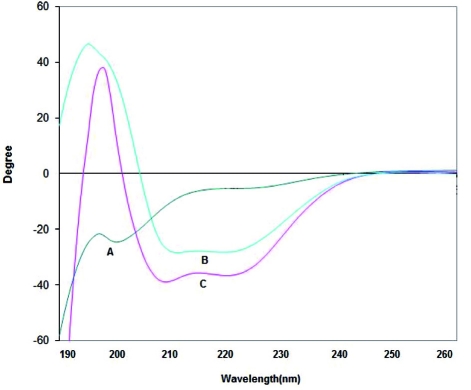
Far-UV circular dichroism spectra obtained from solution of CFP-10 (A), ESAT-6 (B), and ESAT-6/CFP-10 (C).

**Fig. 4:**
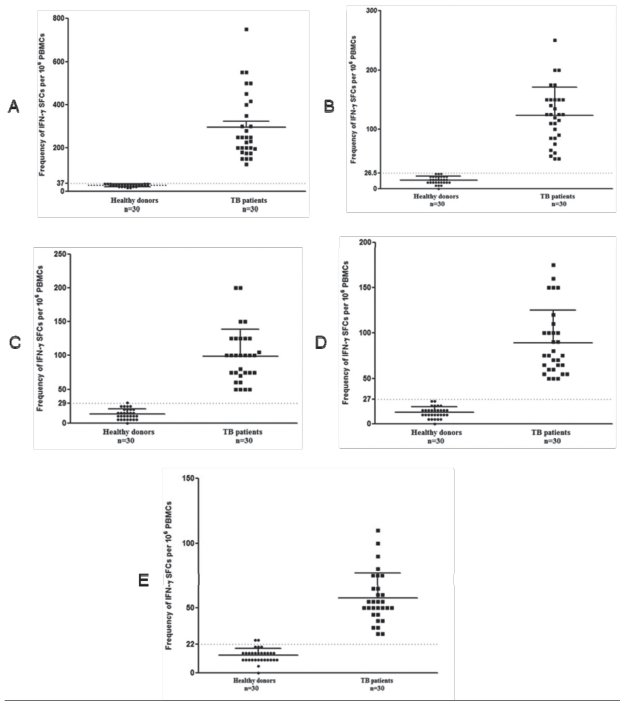
Frequency of IFN-γ SFCs in a million of PBMCs responding to recombinant proteins in different individual groups. A dotted horizontal line is included to show the cutoff value for a positive response for ESAT-6/CFP-10 (A), ESAT-6 (B), CFP-0 (C), commercial ESAT-6 (D), and PPD (E).

The structural states of the recombinant proteins were confirmed by CD spectroscopy. As shown in Figure 3, both ESAT-6 and ESAT-6/CFP-10 have high helical contents, whereas CFP-10 appears to have largely unstructured, random coil structure. The samples from healthy individuals were analyzed to calculate mean and standard deviation (SD) and then cutoff point for positive samples was set at mean + 2SD.6 The distribution of responses to ESAT- 6, CFP-10, ESAT-6/CFP-10, PPD and commercial ESAT-6 was shown in Figure 4. Results are mean values determined by independent observers, given as spots per million PBMCs. Numbers of IFN-γ SFC against ESAT-6, CFP-10 , ESAT-6/CFP-10 and commercial ESAT-6 were significantly higher in TB patients than in BCG-vaccinated healthy donors (Figure 4). All patients participated in this study were previously vaccinated with BCG, nevertheless 25% of them showed negative skin test. All the patients in our IFN-γ ELISPOT analysis for ESAT-6, CFP-10 ESAT-6/CFP-10 and commercial ESAT-6, showed positive reaction. Sensitivity, specificity, positive predictive value (PPV) and negative predictive value (NPV) for recombinant proteins and PPD in ELISPOT assay were summarized in Table 2.

**Table 2:** Values of sensitivity, specificity, PPV and NPV

**Antigens**	**Sensitivity**	**Specificity**	**PPV **	**NPV **
Recombinant ESAT-6	93	100	100	100
Recombinant CFP-10	90	96	96	100
Recombinant ESAT-6/CFP-10	100	100	100	100
Commercial ESAT-6	93	100	100	100
PPD	76	85	90	80

## Discussion

Late diagnosis and treatment of tuberculosis increases the risk of disease dissemination and decreases the survival of some subgroups of patients.[16][17][18] Thus new advancements in techniques, which facilitate rapid diagnosis, are required to control TB successfully. Using ESAT-6 and CFP-10, two specific antigens encoded in the RD1 locus of the bacterial genome, which distinguish M. tuberculosis from other Mycobacteria, has increased specificity and sensitivity of IFN-γ ELISPOT assay.[14] This study reports the results of the first evaluation of ex vivo INF-γ based assay (TSPOTTB) for the diagnosis of TB in adults by recombinant antigens of native M. tuberculosis C as the standard strain of Iran. The antigens chosen for the present study have been found to be strong targets for human B- and T-cell responses.[19] Overlapping peptides of ESAT-6 and CFP-10 offer increased specificity over the PPD skin test when they were used in ELISPOT assay for the diagnosis of M. tuberculosis.[20][21] Arend et al. have shown that ESAT-6 and CFP-10 in combination with T-cell assays can provide the level of specificity and sensitivity necessary to detect exposure to TB.[22] According to our results from ELISPOT assay, these recombinant proteins could discriminate TB patients from healthy BCGvaccinated individuals with p values <0.001. According to values of sensitivity, specificity, PPV and NPV for all recombinant produced proteins, commercial ESAT-6 and PPD, and considering that 25% of TB patients were negative in their TST result, we suggest that ESAT-6 and ESAT-6/CFP-10 proteins could provide high level of confidence for application in diagnosis of TB. Our results indicated higher sensitivity and specificity of fusion protein and poor sensitivity of PPD in the diagnosis of TB patients which is in agreement with other findings.[6][8][20][22] These recombinant proteins offer a realistic and cost effective alternative to PPD and their availability in pure form will facilitate this application. In conclusion, we cloned, expressed and purified large amounts of recombinant secretory antigens ESAT-6, CFP-10 and ESAT- 6/CFP-10. These proteins were purified easily by affinity chromatography and checked with western blotting and circular dichroism methods.

Due to higher sensitivity and specificity, recombinant antigens ESAT-6, CFP-10 and ESAT-6/CFP-10 can be better candidates for detection of TB when compared with conventional diagnostic methods such as PPD skin test. Since there was a high similarity in sensitivity of ELISPOT test between commercial ESAT-6 (a worldwide standard means of TB detection) and produced recombinant proteins, these recombinant antigens can be a reliable diagnostic tool for detection of TB.
